# Q Fever Surveillance in Ruminants, Thailand, 2012

**DOI:** 10.3201/eid1912.130624

**Published:** 2013-12

**Authors:** Samuel L. Yingst, Pattarin Opaschaitat, Reka Kanitpun, Suree Thammasart, Monaya Ekgatat, Vimol Jirathanawat, Preecha Wongwicharn

**Affiliations:** Armed Forces Research Institute of Medical Sciences, Bangkok, Thailand (S.L.Yingst);; National Institute of Animal Health, Bangkok (P. Opaschaitat, R. Kanitpun, S. Thammasart, M. Ekgatat, P. Wongwicharn);; Region 8 Regional Veterinary Diagnostic Center, Surat Thani, Thailand (V. Jirathanawat)

**Keywords:** Q fever, Coxiella burnetii, bacteria, ruminants, surveillance, PCR, placenta, Thailand

**To the Editor:** Two cases of fatal endocarditis in Khon Kaen Province in northeastern Thailand were found to be caused by *Coxiella burnetii* (*1*). Although *C. burnetii* is known to be present in many countries, including in Thailand (*2*), human infection is more commonly associated with sheep and goats, possibly because these animals shed the organism more frequently in vaginal secretions and feces than do large ruminants (*3*).

Surveillance for Q fever, which is caused by *C. burnetii*, in livestock is currently based primarily on serologic or PCR testing of milk (*4*). However, problems in estimating prevalence include serologic assay insensitivity (*5*,*6*) or unavailability of milk from nondairy animals.

For diagnosis of Q fever, the placenta of the animal is commonly tested, but testing is usually conducted only when abortions occur, which is only likely when uninfected animals first encounter *C. burnetii*. Therefore, this approach might underestimate true organism distribution in a disease-endemic area (*7*). In addition, nearly all abortion storms have occurred in sheep or goats, which are rare in Thailand. Ruminant abortion is rarely reported to veterinary authorities in Thailand.

Comparison of paired colostrum and placental samples from sheep showed that *C. burnetii* was found more frequently in placental samples (*8*), which suggested that the placenta is a better sample than milk for surveillance purposes. Also, a placenta may be more useful because it is more likely to contaminate the farm environment. Milk is an unlikely source of Q fever in adult persons because it is seldom consumed by adults in Thailand.

The ideal surveillance strategy would include all relevant samples (serum, milk, and products of conception, both normal and abnormal). However, in practice, cost and logistical limitations dictate refinement of sampling. *C. burnetii* is frequently detected in normal ruminant placentas, but offspring are apparently not affected (*9*). We report that surveillance of normal placentas can provide useful surveillance data.

To test this hypothesis, in 2012 we asked local veterinarians in selected subdistricts in Thailand to contact farmers at their convenience to request that the veterinarians be alerted when a ruminant gave birth. Only grossly normal placentae from normal births of apparently healthy offspring were sampled. Cotyledonary (preferred) or intercotyledonary chorioallantoic tissue was obtained, chilled, and shipped cold to the National Institute for Animal health (Bangkok, Thailand) for analysis. Tissue was ground, extracted, and analyzed by PCR for IS*1111* of *C. burnetii* in a Light Cycler 2.0 Apparatus (Roche, Basel, Switzerland) as described (*10*). To minimize false-positive results, we repeated the PCR with a separate portion of tissue from the original sample. Samples were considered positive if the PCR had a cycle threshold <35 for each assay, or suspected of being positive if this occurred in 1 of 2 separate assays.

Results indicate a high frequency of *C. burnetii* infections in some provinces (Table), which roughly match locations where fatal human cases of endocarditis have occurred (Figure, Appendix). It is common practice among the agrarian population in Thailand to consume ruminant placenta. Although this tissue is reportedly cooked before consumption, the preparation process may result in environmental contamination sufficient to expose persons who were not in close contact with the infected animal.

**Table:** PCR results for Q fever surveillance in ruminants, Thailand, 2012

Province (no. sites)	Animal	No. cases		
		Positive	Negative	Suspected
Chaiyapum (13)	Beef cattle	3	10	0
Chaiyapum (3)	Dairy cattle	1	0	2
Chaiyapum (1)	Goat	0	0	2
Chiang Mai (1)	Buffalo	0	2	2
Kalasin (1)	Goat	1	0	0
Khon Kaen (8)	Beef cattle	8	0	0
Khon Kaen (2)	Buffalo	2	0	0
Maha Sarakham (1)	Goat	2	6	0
Nakon Pathom (9)	Goat	1	9	0
Nakon Ratchasima (1)	Beef cattle	1	1	0
Nakon Ratchasima (17)	Dairy cattle	20	30	0
Nakon Ratchasima (3)	Goat	15	13	0
Prachuap Kiri Khan (9)	Dairy cattle	3	1	6
Ratchaburi (2)	Goat	1	9	0


**Figure:**
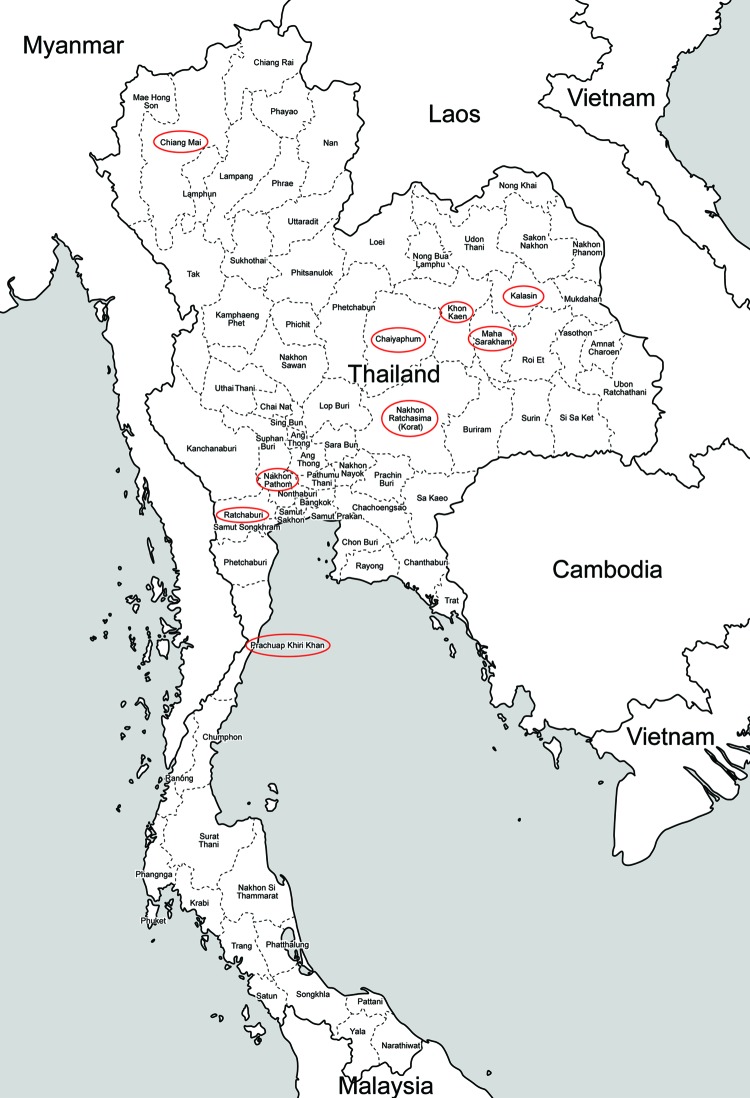
Provinces of Thailand in which Q fever surveillance was conducted, 2012. Red ovals indicate sources of normal ruminant placentas. Two human deaths caused by endocarditis diagnosed as attributable to *Coxiella burnetii* infection have recently been reported in Khon Kaen Province.

This study demonstrates that sampling and PCR of grossly normal ruminant placenta is a viable stand-alone approach for surveillance of *C. burnetii* that might enable the generation, at a minimal cost, of a highly detailed map showing areas where humans and animals are at risk for Q fever. The results indicate that *C. burnetii* is highly endemic in the study region. However, in light of the extreme rarity of serious complications in human infections and lack of any indication of a serious effect on animal production, these results do not indicate a need for veterinary control measures. Nonetheless, food safety practices should be addressed. It is essential that physicians monitoring patients with underlying heart valve conditions encourage such patients to seek diagnosis of any febrile illness so that appropriate treatment may be initiated to minimize risk for complications.

We report a novel approach to Q fever surveillance, which is potentially useful for countries such as Thailand, where subclinical ruminant infections are common. Our results also provide an initial indication of risk factors associated with recent cases of fatal Q fever endocarditis in Thailand. Follow-up research should include broader reservoir species surveillance, environmental surveillance, and comparison of genotypes of organisms found in ruminant placenta with those found in persons with endocarditis. These further efforts will result in clearer understanding of Q fever ecology and potential routes of human exposure.
