# Correction: Vol. 19, No. 12

**DOI:** 10.3201/eid2003.C12003

**Published:** 2014-03

**Keywords:** errata, erratum, correction

In the article Q Fever Surveillance in Ruminants, Thailand, 2012 (S.L. Yingst et al.), the authors incorrectly indicated that the Q fever cases reported in reference 1 (The first reported cases of Q fever endocarditis in Thailand; Infectious Disease Reports; 2012;4:e7; O. Pachirat et al.) were fatal. According to an author of the study, those patients survived. The Yingst article has been corrected online to correctly refer to these cases as “severe”.

