# Correction: Vol. 19, No. 3

**DOI:** 10.3201/eid2007.C12007

**Published:** 2014-07

**Keywords:** errata, erratum, correction

In the article Multidrug-Resistant Tuberculosis, Somalia, 2010–2011 (I. Sindani et al.), author Amal Bassili’s affiliation with the Medical Research Institute, Alexandria University, Alexandria, Egypt, was omitted. The article has been corrected online (http://wwwnc.cdc.gov/eid/article/19/03/12-1287_article.htm).

